# Correlations between measures of executive attention and cortical thickness of left posterior middle frontal gyrus - a dichotic listening study

**DOI:** 10.1186/1744-9081-5-41

**Published:** 2009-10-01

**Authors:** Martin Andersson, Martin Ystad, Arvid Lundervold, Astri J Lundervold

**Affiliations:** 1 Department of Biological and Medical Psychology, University of Bergen, Norway; 2 Kavli's Research Centre for Ageing and Dementia, Haraldsplass Deaconess Hospital, Bergen, Norway; 3 Department of Biomedicine, Neuroinformatics and Image Analysis Laboratory, University of Bergen, Norway; 4 Department of Radiology, Haukeland University Hospital, Bergen, Norway

## Abstract

**Background:**

The frontal lobe has been associated to a wide range of cognitive control functions and is also vulnerable to degeneration in old age. A recent study by Thomsen and colleagues showed a difference between a young and old sample in grey matter density and activation in the left middle frontal cortex (MFC) and performance on a dichotic listening task. The present study investigated this brain behaviour association within a sample of healthy older individuals, and predicted a positive correlation between performance in a condition requiring executive attention and measures of grey matter structure of the posterior left MFC.

**Methods:**

A dichotic listening forced attention paradigm was used to measure attention control functions. Subjects were instructed to report only the left or the right ear syllable of a dichotically presented consonant-vowel syllable pair. A conflict situation appears when subjects are instructed to report the left ear stimulus, caused by the conflict with the bottom-up, stimulus-driven right ear advantage. Overcoming this processing conflict was used as a measure of executive attention. Thickness and volumes of frontal lobe regions were derived from automated segmentation of 3D magnetic resonance image acquisitions.

**Results:**

The results revealed a statistically significant positive correlation between the thickness measure of the left posterior MFC and performance on the dichotic listening measures of executive attention. Follow-up analyses showed that this correlation was only statistically significant in the subgroup that showed the typical bottom-up, stimulus-driven right ear advantage.

**Conclusion:**

The results suggest that the left MFC is a part of an executive attention network, and that the dichotic listening forced attention paradigm may be a feasible tool for assessing subtle attentional dysfunctions in older adults.

## Background

Performance on tasks involving cognitive control functions, such as managing interference, sustained attention and suppression of habitual responses, has been associated with frontal lobe functions [1-5]. Since aging is associated with a reduction of frontal lobe grey matter volume [6,7] and cortical thickness [8] as well as a decline in cognitive control functions [1,9-13], it is reasonable to expect a correlation between these structural and behavioural changes in elderly subjects. In the present study we investigated this hypothesis in a sample of healthy older adults by using structural Magnetic Resonance Imaging (MRI) measures of segmented frontal lobe regions and a forced-attention dichotic listening procedure [14] to measure cognitive control functions [15].

In a dichotic listening situation a subject is presented with two different syllables, one to each ear simultaneously, with instruction to report the syllable heard best. The typical result is more correct reports from the right compared to the left ear ([16]. This right ear advantage (REA) effect has been explained by two models: the structural model and the attentional model [17]. The structural model explains the right ear advantage effect as a consequence of the wiring of the brain's auditory neural pathways and the left hemisphere specialization for speech sound processing, whereas the attentional model assumes that the anticipation of verbal material primes the left hemisphere which arouses the left hemisphere and directs attention to the contralateral right side (for an overview see [17]). When the subjects are instructed to report stimuli presented to the right ear in a forced-right (FR) condition, the bottom-up processing and the instruction to report the right ear stimuli are working synergistically, both facilitating a right ear report [15,18,19]. On the other hand, in a forced-left (FL) condition the subjects must control the bottom-up stimulus driven right ear dominance in order to report the left ear stimuli. Overcoming this processing conflict is suggested by Hugdahl and colleagues [15] to require an executive cognitive control process that includes requirements for executive attention, defined as an ability to maintain information in the presence of interference or prevent attentional focus from distraction ([20,21]).

Previous functional Magnetic Resonance Imaging (fMRI) studies have shown that performance on a dichotic listening task involves the frontal lobe [22-24], and that prefrontal activation in the forced-left condition is increased when contrasted to the activation pattern obtained during the forced-right condition [25]. Moreover, a combined functional and structural MRI study showed both reduced activation in the forced left condition and reduced density of grey matter in the left middle frontal cortex (MFC) in an older compared to a younger group of subjects [26]. The location of the reduced grey matter reported by these authors is within the caudal or posterior part of the left MFC region as segmented by the FreeSurfer software (see method section below). The present study included a larger sample of elderly subjects than in Thomsen et al.'s study, a more comprehensive image segmentation procedure where MRI volumes of frontal lobe regions were normalized by the intracranial volume (ICV) to account for head size, and where intra-individual brain and behaviour associations were investigated by correlation analyses. We predicted a positive correlation between the Dichotic listening measures of executive attention and measures of the frontal lobe grey matter volume and thickness of the posterior left MFC. Secondly, we examined the specificity of this particular correlation, and examined if the dichotic listening measures correlated with several other cortical frontal lobe regions. Third, in that the conflict situation of the FL condition is related to a right-ear processing advantage [15], we expected that the correlations would be higher in subjects showing a right ear advantage (REA) than in subjects with a left ear or no ear advantage (LEA/NEA) in a non-forced condition.

## Methods

### Subjects

The subjects were part of a longitudinal study of cognitive aging, including a neuropsychological examination and genetic testing (N = 163), and a subsample was also examined with MRI. Subjects with previous or present neurological diseases and psychiatric disorders, history of substance abuse, or hearing loss were excluded. The 71 subjects with post processed MRI data who also performed the Dichotic listening task were included in the present study. The subjects performed the Dichotic listening task the same day as they were scanned. The included subjects' cognitive function was evaluated as normal according to an overall evaluation of the neuropsychological test results. The neuropsychological examination included tests assessing verbal and visual memory, auditory and visual attention functions, processing speed, verbal fluency, intelligence and tests of executive functions as well as an odour identification test. The MRIs were evaluated by an experienced neuroradiologist, and two subjects with pathological findings were excluded.

The final sample included 69 subjects, 50 females and 19 males, with a mean age of 58.6 yrs. (SD = 7.1). Their mean years of education was 14.1 (SD = 3.1) and their mean estimated IQ (Matrices, Vocabulary; from Wechsler's Abbreviated Scale of Intelligence) was 114.8 (SD = 11.2). There were no statistically significant differences between the males and females with regard to IQ, years of education and age (independent t-tests, all p > .05). The four left-handed subjects (one male) were not statistically significant different from the right-handed subjects regarding age, IQ and years of education (independent t-test, all p > .05). The study was approved by the Regional Committee for Medical Research Ethics, Southern Norway.

### The Dichotic listening paradigm

The dichotic listening stimuli consisted of the six consonant-vowel syllables/ba/da/ga/pa/ta/ka/that were presented pair-wise to the subjects, with one syllable presented to the right ear while another syllable was simultaneously presented to the left ear, thus forming 36 possible dichotic combinations. The six homonymic pairs were included as test trials and were excluded from the statistical analyses. The number of correct reports from left and right ear was registered separately, and the maximum score was 30 for each ear (right, left) and condition (NF, FR, FL; see below). The syllables were recorded with a male voice with constant intonation and intensity and stored on a PC. A simultaneous onset for the left and right ear channel was enabled by using the Goldwave [27] audio editing software. Mean duration of the syllables was 350-400 ms, and the inter trial interval was approximately four seconds. The 36 dichotic pairs were recorded three times on a CD with different randomizations, one for each condition, thus giving a total of 108 presentations. The stimuli were presented to the subjects through so-called "plug-in type earphones" from a standard CD player, with an extra set of headphones connected so that the experimenter could listen to the presentations. The report made by the subjects on each trial (requiring a single response) was registered on a special scoring form. Stimulus material, test and scoring procedures followed the general descriptions given in [15,28,29]. The subjects were shown a sheet with the syllables written in large fonts, and the subjects were required to read the syllables aloud to the experimenter before the test was administered. This was done to familiarize the subjects with the syllables, and to ensure that they knew their correct pronunciations. The 36 syllable pairs were presented three times, one for each of three Dichotic listening conditions: the non-forced, forced-right and the forced-left condition.

In the non-forced (NF) condition, the subjects were instructed to report the syllable they heard best or most clearly after each dichotic presentation. Before the testing, all subjects were presented with a few practice trials. The intensity level of the consonant-vowel syllable presentations was individually adjusted, based on feedback from the participants.

In the forced-right (FR) condition the subjects were instructed to listen to the right ear and to only report the syllables presented to the right ear. Otherwise the procedure and stimulus presentations were identical as for the NF condition. In the forced-left (FL) condition, the only difference from the FR condition was that the subjects were instructed to listen to the left ear and only report the syllable presented to the left ear. In the forced conditions the experimenter pointed to and named their right ear in the FR and their left ear in FL condition, and placed a marker (a cubic pen-sharpener) to the right side on the desk in front of the subject in the FR condition, and to the left side in the FL condition. The NF condition was always presented first since it may be difficult to unattend once given an instruction to direct attention to either the right or left ear. The presentation order (i.e. FR before the FL condition, or FL before the FR condition) of the two forced instruction conditions was randomized.

### Measures of attention control

The number of correct reports from the left (LE) and right (RE) ear and a laterality index (LX = 100(RE-LE)/(RE+LE)) will be reported from all three Dichotic listening conditions (NF, FR, FL). The number of LE reports and the laterality index score from the FL condition were used as measures of executive attention. This was based on a model of Dichotic listening performance [15] suggesting that the instruction to report LE syllables leads to a conflict situation where the subjects must overcome a more automatic or bottom-up, stimulus-driven right ear advantage. In this conflict situation, the subjects must rely on an executive cognitive control process comprising what Engle [20] referred to as "executive attention" [15], a term that has been defined as an ability to maintain information in the presence of interference or prevent attentional focus from distraction ([20,21]).

### MR protocol

The MRI scans were acquired on a 1.5 T GE Sigma Echospeed scanner with a standard 8-channel head coil, using 256 × 256 × 124 dual-volume saggital T1-weighted 3D FSPGR IR prepared acquisitions (TR/TE/TI/FA = 9.5/2.2/450/7°) at voxel-size 0.94 × 0.94 × 1.4 mm^3^. The resulting DICOM images were then transferred to a local Linux workstation for further processing as described below.

### MRI morphometric analyses

The FreeSurfer [30] analysis software tool was used to analyse the MR data. In order to improve signal-to-noise ratio, eliminate movement artefacts, and thus to obtain at better representation of the actual brain, two consecutive scans were acquired. The two T1 weighted volumes were then coregistered and averaged before skull stripping [31], normalization, and Talairach conversion were performed [32]. Volumetric measurement of the cortical sheet through the automated procedures is described in Salat et al. [8]. Cortical thickness measures were obtained by reconstructing the grey/white matter boundary surface and the gray/pial boundary surface and calculating the distance between these two surfaces at each point on the cortical sheet. This particular segmentation method applies both signal intensity and geometric and topological information from the entire 3D-MRI volume. As thickness measurements are not dependent on the original voxel resolution of the images, the geometrically reconstructed tissue boundary surfaces can be used to detect sub-millimeter differences between groups.

**Figure 1:**
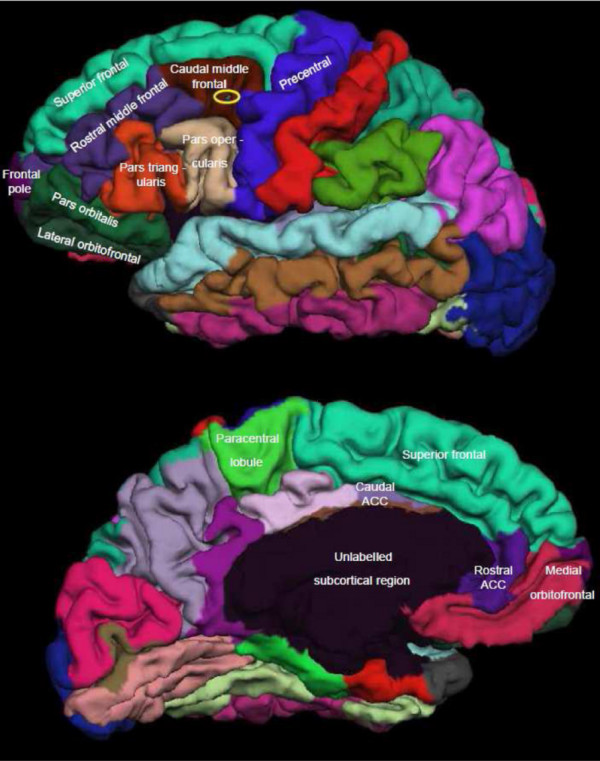
**Frontal regions**. shows the lateral (above) and the medial view (below) of frontal lobe segmented regions in a male subject in his 60 ties. All FreeSurfer segmented frontal lobe regions described in the text is also labelled in Figure 1. The position of the Thomsen et al. (2004b) report of reduced grey matter density for the old group was when compared to FreeSurfer regions within the caudal (or posterior) left middle frontal cortex (MFC), and the location is illustrated with a yellow ellipse on the lateral view in Figure 1.

The frontal lobe was segmented into several regions of interest by the FreeSurfer processing stream, with separate measures for grey matter volume and cortical thickness of gyral regions in each hemisphere. The frontal lobe regions that were used in the morphometric analyses were for each hemisphere as follows: the superior frontal cortex, middle frontal cortex (MFC) (caudal middle frontal, rostral middle frontal), inferior frontal cortex (IFC) (pars opercularis, pars triangularis, pars orbitalis), orbitofrontal cortex (OFC) (lateral orbitofrontal, medial orbitofrontal), frontal pole, precentral, and the paracentral lobule [33]. In addition, cortical thickness and grey matter volumes were obtained also for the left and right anterior cingulate cortex (ACC) (caudal anterior cingulate, rostral anterior cingulate) since the ACC is found to be associated with cognitive control tasks [34-36]. Figure 1 shows the frontal lobe segmented regions taken from a single subject from the study. The position of the reduced grey matter reported by Thomsen et al. [26] is illustrated in Figure 1 on the lateral view.

### Statistical analyses

A repeated measures analysis of variance on the design Condition (NF, FR, FL) × Ear (right, left) was computed to analyze the Dichotic listening results, and correlation analyses were used to investigate associations between the behavioural measures of attention control and MRI measures of frontal lobe structures. Due to multiple comparisons in the correlation analyses, the significance threshold was set to p < .01. Since frontal brain volume measures may be influenced by differences in total brain size, FreeSurfer derived estimates of intra-cranial volume (ICV) were included. Linear regression models were run to normalize the volume and thickness measures on ICV. The standardized residuals of the MRI measures were extracted by removing the variance shared by the ICV measure, using the ICV as a predictor variable and each of the structural measures as outcome variables. The resulting standardized residuals were used in the subsequent correlation analyses. This procedure has been used in earlier studies of neuroanatomical aspects of aging [37]. Correlations based on the non-adjusted MRI measures were also computed.

To answer the primary question, separate correlation analyses were run between the cortical thickness and grey matter volume measures of the posterior left MFC and the Dichotic listening measures (i.e. the number of correct left and right ear reports and the laterality index).

To investigate if the Dichotic listening measures were correlated with several frontal lobe structures, the cortical thickness and volume areas (each n = 14) for the left and right frontal hemisphere were included in separate analyses.

Significant correlations were followed up by separate correlation analyses for subjects who showed a right ear advantage (n = 47) and for subjects who showed a left ear or no ear advantage (n = 22) in the NF condition (p < .05). Finally, statistically significant correlations were re-analysed as partial correlations in order to control for possible confounding effects of age, sex, estimated IQ, and education (p < .01).

## Results

### The Dichotic listening task

**Figure 2:**
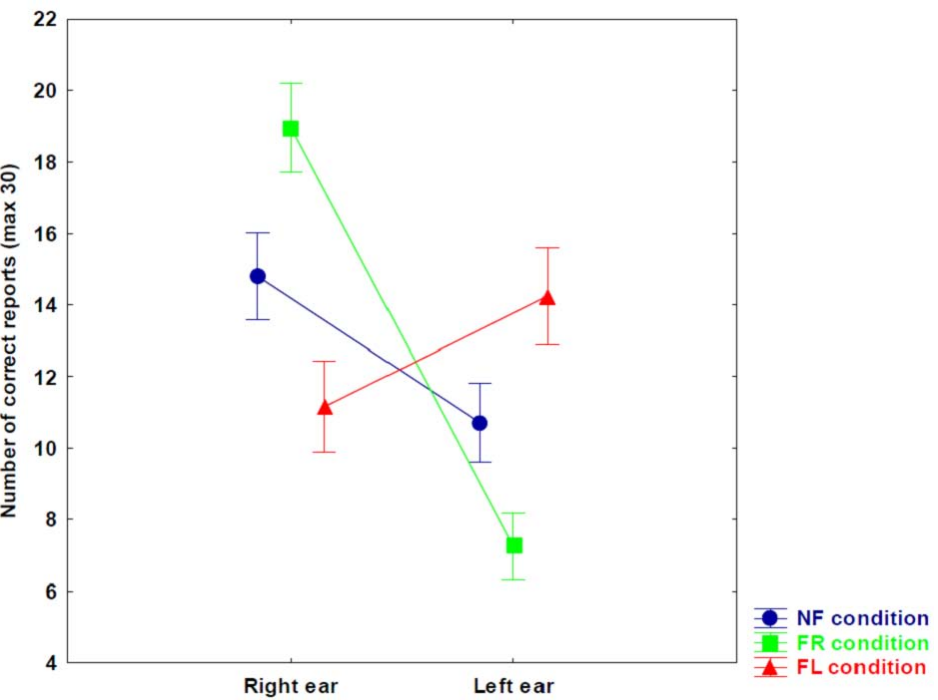
**Dichotic listening**. NF denote non-forced, FR denote forced right, and FL denote forced left condition. Vertical bars denote 0.95 confidence intervals.

The Dichotic listening results showed a statistically significant main-effect of Ear, F(1,68) = 27.951, *p *< 0.001, caused by overall more correct reports from the right (RE mean = 14.97, SD = 3.8) than the left ear (LE mean = 10.73, SD = 3.3). There was also a statistically significant two-way interaction between Ear × Condition, F(2,136) = 56.167, *p *< .001, with a statistically significant right ear advantage in the NF and FR conditions and a statistically significant left ear advantage in the FL condition (*p *< .05) (see Figure 2 for means).

### Posterior left MFC and executive attention

**Figure 3:**
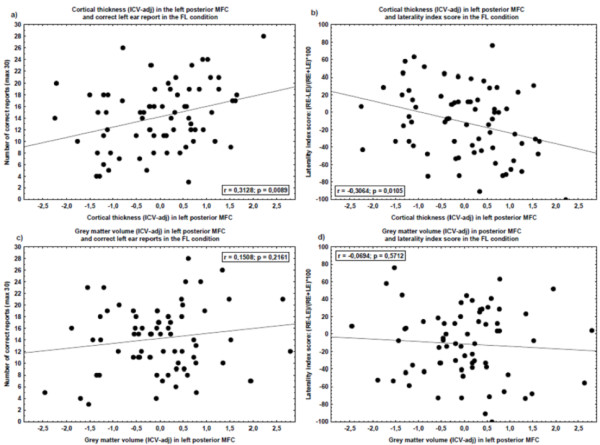
**Scatterplots**. The scatter-plots show measures of grey matter of left posterior middle frontal cortex (MFC) (x-axes) and performances on the forced left (FL) condition (y-axes). Each dot represents one subject. The left posterior MFC volume and thickness measures are reported as standard residuals after the influence of intra-cranial volume (ICV) was removed (linear regression). The two scatter-plots above show the cortical thickness in the left posterior MFC to a) number of correct left ear report on FL condition (left scatter-plot), and b) a laterality index score ((RE-LE)/(RE+LE)*100) in the FL condition (right scatter-plot). The two scatter-plots below show the grey matter volume in the left posterior MFC to c) number of correct left ear report on FL condition (left scatter-plot), and d) a laterality index score ((RE-LE)/(RE+LE)*100) in the FL condition (right scatter-plot).

The correlation analyses between the ICV adjusted cortical thickness and volume in the posterior left MFC and Dichotic listening performance showed a statistically significant positive correlation between cortical thickness in the posterior left MFC and the number of correct LE reports in the FL condition (r = .31, p = .009). Statistically significant correlations were also found between the ICV-unadjusted measure of the cortical thickness of the posterior left MFC and the number of correct LE reports in the FL condition (r = .32, p = .007) as well as the FL laterality index score (r = -.32, p = .006). Figure 3 shows scatterplots of the correlations between measures of grey matter in left posterior MFC (adjusted of ICV) and Dichotic listening measures of executive attention.

### Frontal lobe structures and executive attention

Correlation analyses including grey matter volume and cortical thickness of all frontal lobe regions and the Dichotic listening performance showed one additional significant correlation. The number of correct LE reports in the FR condition was significantly correlated with both the ICV adjusted and non-adjusted grey matter volume in the posterior part of the left anterior cingulate cortex (r = .31, p = .009). No other correlations were statistically significant (*p *> .01). (See additional file 1: Cortical thickness, standardized residuals; and additional file 2: Grey matter volume, standardized residuals).

### Subjects with right ear advantage and left ear or no ear advantage in the NF condition

Statistically significant correlations between the left posterior MFC and the FL condition were re-computed in the right ear advantage and left ear or no ear advantage subsamples, and were only confirmed in the right ear advantage subgroup (See additional file 3: Subjects divided into right ear advantage (REA) and left ear or no ear advantage (LEA/NEA) subgroups). The ICV adjusted thickness-measure of the posterior left MFC showed a statistically significant positive correlation with the LE reports in the FL condition (r = .43, p = .003), and statistically significant negative correlations with the FL laterality index score (r = -.42, p = 004) and the number of FL RE reports (r = -.35, p = .017)). The correlation results were also statistically significant when including the unadjusted thickness-measure of the posterior left MFC and the LE reports in the FL condition (r = .43, p = .002), the FL laterality index score (r = -.43, p = .002) and the number of FL RE reports (r = -.36, p = .012). No other correlation was statistically significant (*p *> .05).

Finally, partial correlations were computed on the statistically significant correlations shown in the right ear advantage subgroup in order to control for possible confounding variables (i.e. age, sex, education and estimated IQ). The results showed that all statistically significant correlations, except for the FL RE reports, remained statistically significant (*p *< .01) (See additional file 4: Controlling for confounding variables).

## Discussion

The results showed significant correlations between Dichotic listening measures of executive attention and structural measures of the posterior left MFC. These correlations were found for cortical thickness measures, but not for the volumetric measures. The results were moreover significant for both ICV adjusted and unadjusted thickness measures. The load on attentional control during the FL condition was expected to be largest in participants showing a right ear advantage in the NF condition, due to the conflict between such a processing advantage and the instruction to report from the left ear [15]. The present results support this hypothesis by showing that the correlations between the Dichotic listening measures of executive attention and the MRI measures of the posterior left MFC were only statistically significant in the participants that have such a right ear advantage in the NF condition. In other words, assuming that the degree of cognitive performance is associated with volume size of measured tissue being involved, the results indicate that the left posterior MFC is involved in a task that requires executive attention. Executive attention, defined as the ability to maintain information in the presence of interference or prevent attentional focus from distraction [20,21], is suggested to be required in the FL condition [15].

The current findings support previous reports of a particular role of the left MFC when performing the FL instruction condition part of a Dichotic listening task [25,26]. In Thomsen et al.'s study [26] this was shown by comparing differences in grey matter density between participants in a young (N = 13) and in an old (N = 8) sample. The present study extended this finding by revealing a direct correlation between behavioural and structural measures within a sample of older adults. Furthermore, using another MRI segmentation method, not based on the concept of voxel-based morphometry, our results can be interpreted more directly in terms of mean cortical thickness within labelled cortical patches. The parcellation of the cortex is automated and based on a probabilistic approach comparing the current dataset with an atlas. The method has been proven to be comparable in accuracy to manual labelling [38]. This method may explain why thickness rather than volumetric measures were correlated with the Dichotic listening measures of executive attention, because thickness measures are expected to be more robust than volume-measurements of the parcellated cortical subregions. The parcellations of the cortical areas are influenced by the shape of the cortex. The extent or area coverage of the labelled region strongly influences the volume measure, but not so much the thickness measure. This is because the thickness measure is the distance between the grey/white matter boundary surface and the gray/pial boundary surface only, whereas a measure of volume also is highly dependent on the area coverage of the parcellated subregions. We suggest that since segmentation dependent variability in regional cortical volumes might be larger than cortical thickness variation within the same regions, our statistical significant findings only hold for the latter.

We expected that brain morphometric results could be influenced by the ICV of the subjects. Although frontal structures are known to be reduced by age, the ICV measure is known not to change with age. This claim was supported since there was no correlation between age and ICV in the present study (r = -.087, p = .48). However, the specificity of the correlation between measures of the executive attention and the posterior part of the left MFC was confirmed both by using MRI measures that were adjusted and non-adjusted for ICV. We will argue that this strengthens the robustness of the present findings.

The main strength of the present study was the use of automated procedures for segmenting the MRI data, reducing the subjectivity of volumetric analyses that may have been a problem in earlier studies. An additional strength is the inclusion of healthy elderly adults that performed a relatively simple task, with the single manipulation of the direction of attention focus to either the left or right side in auditory space. This manipulation was moreover assumed not to be heavily influenced by previous general knowledge and accumulated experience. The requirement for only single syllable report is also considered to minimize the demands on cognitive function (i.e. short-term memory) [28]. The present results may thus reveal a quite specific and subtle effect that may not be detected by using more complex tasks. Several studies have shown a reduction in the ability to modulate the ear advantage for older compared to younger adults when instructed to report the left ear stimulus in a FL condition [15,26,39-41] (see also [16,42]). However, longitudinal studies are necessary to investigate the validity of the forced attention Dichotic listening paradigm as a marker for cognitive decline.

### Limitations

While the results showed a statistically significant correlation between measures of executive attention and the left posterior MFC, it is also important to note that there are large individual differences (cf. Figure 3a). Moreover, although subjects with reported hearing loss were excluded, the subjects did not go through a comprehensive audiometrical examination. This is a limitation since it has been shown that dichotic listening performances differ for old subjects with sensorineural loss [43]. Another limitation was related to the fact that the examination only included morphometric measures of frontal lobe structures. Other structures, such as the temporal and parietal lobes [17,44] and white matter structures in the corpus callosum [45], have also been associated with Dichotic listening performance.

## Conclusion

The present study showed a statistically significant correlation between performance in the FL condition and a specific part of the prefrontal area in a sample of healthy elderly subjects. This was interpreted with reference to a forced attention model [15], suggesting that performance in the FL condition is dependent on executive attention functions, or what Kane and Engle [20,21] described as an ability to maintain attention focus in the presence of interference. In conclusion, we suggest that the posterior left MFC may have an important role in tasks requiring an executive attention function, and that the forced attention Dichotic listening paradigm may be a feasible tool for assessing subtle attentional dysfunctions associated with cognitive aging.

## Competing interests

The authors declare that they have no competing interests.

## Authors' contributions

MA: Neuropsychological testing, statistical analysis, main author. MY: Morphometric analysis and internal review. AL: MRI acquisition protocol design, morphometric analysis and internal review. AJL: Study design, supervisor, internal review.

All authors have read and approved the final manuscript

## Supplementary Material

Additional file 1 **Cortical thickness, standardized residuals**. RE denotes number of correct right ear reports, and LE denotes number correct of left ear reports. LX denote a laterality index score ((RE-LE)/(RE+LE) * 100). Cortical thickness values denote average distance between white/grey matter boundary and the grey/pial matter boundary in millimetres, after the influence of ICV is regressed out. The analyses were made with Pearson correlations. All correlations are 2-tailed, and correlations significant at *p. *< 0.01 are marked with asterisk and printed in bold. Click here for file

Additional file 2 **Grey matter volume, standardized residuals**. RE denotes number of correct right ear reports, and LE denotes number correct of left ear reports. LX denote a laterality index score ((RE-LE)/(RE+LE) * 100). Gray matter volume standardized residual denotes the mm3 after the influence of ICV is regressed out. The analyses were made with Pearson correlations. All correlations are 2-tailed, and correlations significant at *p. *< 0.01 are marked with asterisk and printed in bold. Click here for file

Additional file 3 **Subjects divided into right ear advantage (REA) and left ear or no ear advantage (LEA/NEA) subgroups **. RE denotes number of correct right ear reports, and LE denotes number correct of left ear reports. LX denotes a laterality index score ((RE-LE)/(RE+LE) * 100). Cortical thickness values denote average distance between white/grey matter boundary and the grey/pial matter boundary in millimetres. Measures of grey matter are shown both adjusted for ICV (stand. residuals) and unadjusted for ICV (unadj). The analyses were made with Pearson correlations. All correlations are 2-tailed, and correlations significant at *p. *< 0.05 are marked with asterisk and printed in bold. Click here for file

Additional file 4 **Controlling for confounding variables **. RE denotes number of correct right ear reports, and LE denotes number correct of left ear reports. LX denotes a laterality index score ((RE-LE)/(RE+LE) * 100). Cortical thickness values denote average distance between white/grey matter boundary and the grey/pial matter boundary in millimetres. Measures of grey matter are shown both adjusted for ICV (stand. residuals) and unadjusted for ICV (unadj). The analyses were made with partial correlations. All correlations are 2-tailed, and correlations significant at *p. *< 0.01 are marked with asterisk and printed in bold. Click here for file
