# Supplementary figures and images for: Correlations between measures of executive attention and cortical thickness of left posterior middle frontal gyrus - a dichotic listening study
Source: Behav Brain Funct. 2009 Oct 1;5:41. doi: 10.1186/1744-9081-5-41 (PMC2761925; doi:10.1186/1744-9081-5-41)

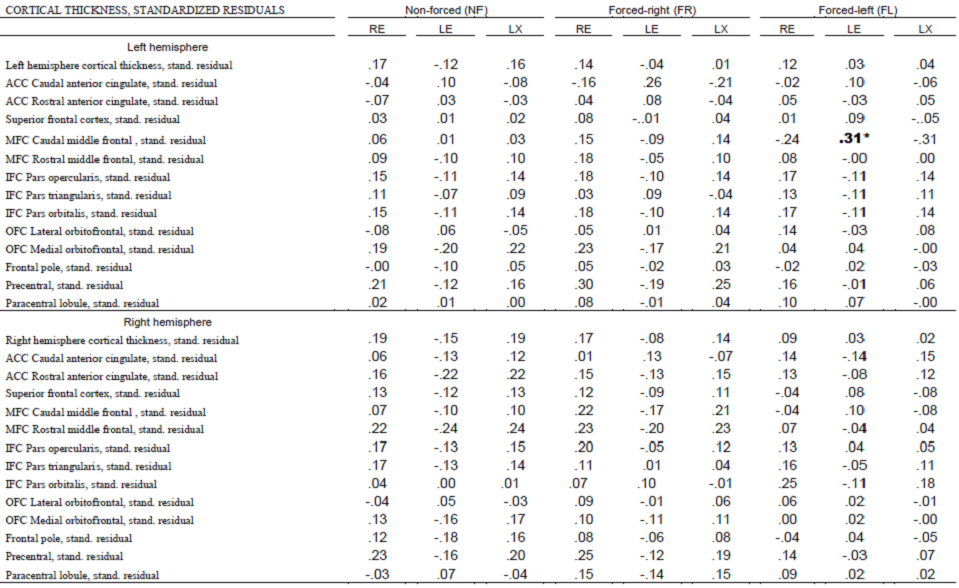

Supplement: Additional file 1 — Cortical thickness, standardized residuals. RE denotes number of correct right ear reports, and LE denotes number correct of left ear reports. LX denote a laterality index score ((RE-LE)/(RE+LE) * 100). Cortical thickness values denote average distance between white/grey matter boundary and the grey/pial matter boundary in millimetres, after the influence of ICV is regressed out. The analyses were made with Pearson correlations. All correlations are 2-tailed, and correlations significant at p. < 0.01 are marked with asterisk and printed in bold. [file 1744-9081-5-41-S1.BMP]

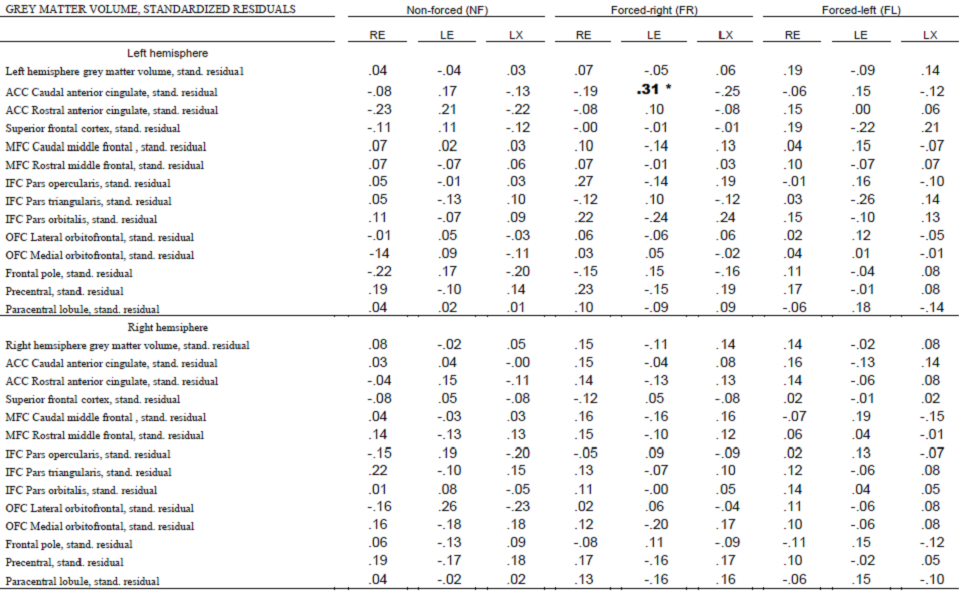

Supplement: Additional file 2 — Grey matter volume, standardized residuals. RE denotes number of correct right ear reports, and LE denotes number correct of left ear reports. LX denote a laterality index score ((RE-LE)/(RE+LE) * 100). Gray matter volume standardized residual denotes the mm3 after the influence of ICV is regressed out. The analyses were made with Pearson correlations. All correlations are 2-tailed, and correlations significant at p. < 0.01 are marked with asterisk and printed in bold. [file 1744-9081-5-41-S2.BMP]

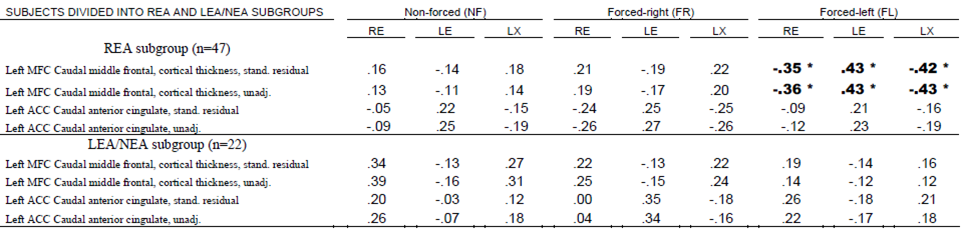

Supplement: Additional file 3 — Subjects divided into right ear advantage (REA) and left ear or no ear advantage (LEA/NEA) subgroups . RE denotes number of correct right ear reports, and LE denotes number correct of left ear reports. LX denotes a laterality index score ((RE-LE)/(RE+LE) * 100). Cortical thickness values denote average distance between white/grey matter boundary and the grey/pial matter boundary in millimetres. Measures of grey matter are shown both adjusted for ICV (stand. residuals) and unadjusted for ICV (unadj). The analyses were made with Pearson correlations. All correlations are 2-tailed, and correlations significant at p. < 0.05 are marked with asterisk and printed in bold. [file 1744-9081-5-41-S3.BMP]

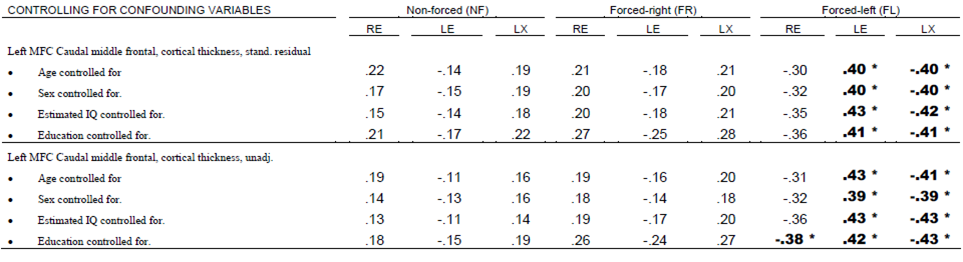

Supplement: Additional file 4 — Controlling for confounding variables . RE denotes number of correct right ear reports, and LE denotes number correct of left ear reports. LX denotes a laterality index score ((RE-LE)/(RE+LE) * 100). Cortical thickness values denote average distance between white/grey matter boundary and the grey/pial matter boundary in millimetres. Measures of grey matter are shown both adjusted for ICV (stand. residuals) and unadjusted for ICV (unadj). The analyses were made with partial correlations. All correlations are 2-tailed, and correlations significant at p. < 0.01 are marked with asterisk and printed in bold. [file 1744-9081-5-41-S4.BMP]
